# Characterization of Transcriptomic and Proteomic Changes in the Skin after Chronic Fluocinolone Acetonide Treatment

**DOI:** 10.3390/biom12121822

**Published:** 2022-12-06

**Authors:** Yongsu Choi, Masaki Takasugi, Kazuaki Takemura, Yuya Yoshida, Tomonori Kamiya, Jun Adachi, Daisuke Tsuruta, Naoko Ohtani

**Affiliations:** 1 Department of Pathophysiology, Graduate School of Medicine, Osaka Metropolitan University, Osaka 545-8585, Japan; 2 Department of Dermatology, Graduate School of Medicine, Osaka Metropolitan University, Osaka 545-8585, Japan; 3 Laboratory of Proteomics for Drug Discovery, Center for Drug Design Research, National Institute of Biomedical Innovation, Health and Nutrition, Ibaraki City 567-0085, Japan

**Keywords:** topical corticosteroids (TCS), fluocinolone acetonide (FA), transcriptomics, proteomics

## Abstract

While topical corticosteroid (TCS) treatment is widely used for many skin diseases, it can trigger adverse side effects, and some of such effects can last for a long time after stopping the treatment. However, molecular changes induced by TCS treatment remain largely unexplored, although transient changes in histology and some major ECM components have been documented. Here, we investigated transcriptomic and proteomic changes induced by fluocinolone acetonide (FA) treatment in the mouse skin by conducting RNA-Seq and quantitative proteomics. Chronic FA treatment affected the expression of 4229 genes, where downregulated genes were involved in cell-cycle progression and ECM organization, and upregulated genes were involved in lipid metabolism. The effects of FA on transcriptome and histology of the skin largely returned to normal by two weeks after the treatment. Only a fraction of transcriptomic changes were reflected by proteomic changes, and the expression of 46 proteins was affected one day after chronic FA treatment. A comparable number of proteins were differentially expressed between control and FA-treated skin samples even at 15 and 30 days after stopping chronic FA treatment. Interestingly, proteins affected during and after chronic FA treatment were largely different. Our results provide fundamental information of molecular changes induced by FA treatment in the skin.

## 1. Introduction

Glucocorticoids (GCs) are a class of corticosteroids and regulate body homeostasis including immune response and metabolism, mainly by modulating gene expression through binding to glucocorticoid receptors (GRs) [1]. The application of topical corticosteroids (TCSs) such as glucocorticoid fluocinolone acetonide (FA) is the basic therapy for many skin diseases including atopic dermatitis and psoriasis. Side effects of TCSs include skin thinning, fragility, and permanent striae [1,2]. Side effects of TCSs are usually but not always completely reversible, which could be clinically problematic [1,3]. Moreover, the side effects of TCSs are sometimes hardly tolerated, especially when causing long-lasting dermatitis [4]. The molecular mechanisms underlying the adverse effects of TCSs are currently poorly understood. Moreover, molecular changes induced by TCSs still remain largely unexplored. Although many studies described how TCS treatment on the skin induces transient changes in representative ECM components and histology, its effects on the transcriptome and proteome of the skin after the treatment have not been well investigated [5,6,7,8,9,10,11,12]. In this study, we showed how mRNA and protein expression change after TCS treatment. Although TCS-treated skin reverted to normal histology by 15 days, the expression of some genes, including those with known importance in skin physiology, kept changing even 4 weeks after TCS treatment.

## 2. Materials and Methods

### 2.1. Animal Experiments

Female C57BL/6J mice were purchased from CLEA Japan. At 7 weeks old, dorsal hair was shaved and FA (5 µg in 200 µL acetone) or vehicle were topically applied once every 3 days for 12 days. The dorsal skin was collected 1, 15, or 30 days after stopping the treatment and was processed for histological, RNA, or protein analysis. The mice were maintained under specific pathogen-free conditions on a 12 h light–dark cycle. All procedures were approved by the Institutional Animal Care and Use Committee at Osaka Metropolitan University.

### 2.2. Histological Analysis and Immunohistochemistry

The dorsal mouse skin samples in each time point were collected, and the skin samples were fixed with 4% paraformaldehyde. After washing with tap water for 30 min, the tissue samples were dehydrated with 70% ethanol and embedded in paraffin. The samples were cut by a microtome with a 4 µm thickness and mounted on slide glass. Paraffin was removed, and the slides were stained with hematoxylin and eosin (HE). The thickness of the skin was measured at more than 5 points and the number of cells was counted for 2 fields of views for each sample. Ki67 immunohistochemistry was performed using anti-Ki67 antibody (Invitrogen, SP6, Waltham, MA, USA) and DAB detection kit (Takara, MK210, Tokyo, Japan). Counterstaining was performed with HE. Cells were counted at least for four fields of view for each sample and Ki67 positive ratio was calculated as the number of Ki67 positive cells/epithelial cells (excluding hair follicle cells).

### 2.3. RNA Extraction

The dorsal skin samples collected from mice was immediately frozen in liquid nitrogen and was sliced into 16 µm sections using a cryostat in order to promote the permeation of RNA isolation reagent. Prompt permeation of RNA isolation reagent that inactivates RNase is crucial for purifying intact RNA especially from the skin [13]. RNA was extracted using RNAiso Plus reagent (Takara).

### 2.4. Transcriptomic Ananlysis

Libraries were prepared using an NEBNext Ultra II Directional RNA Library Prep Kit with the NEBNext Poly(A) mRNA Magnetic Isolation Module (New England Biolabs, Ipswich, MA, USA). Libraries were sequenced on NextSeq 500 with a single-end 75 bp run. Raw reads were trimmed using fastp and then mapped to the mouse genome (GRCm39) using Hisat2. Read counts per gene were obtained using featureCounts. Normalization and statistical analysis were performed using DESeq2 [14]. Raw reads have been deposited in the ArrayExpress database under the accession number E-MTAB-11992. Ontology and TF target enrichment analysis was performed using Enrichr [15], and the results were visualized using REViGO [16].

### 2.5. Real-Time qPCR

Total RNA was reverse transcribed into cDNA with PrimeScript (Takara) and real-time qPCR was performed using TB Green Premix Ex Taq II (Takara) with StepOne Plus Real Time PCR system (Thermo Fisher Scientific, Waltham, MA, USA). The primer sequences used in this study were as follows: *Gapdh*, 5′-CAACTACATGGTCTACATGTTC-3′ (forward) and 5′-CGCCAGTAGACTCCACGAC-3′ (reverse); and *Fkbp5*, 5′-GTGGGTTCTACATCGGCACT-3′ (forward) and 5′-GAGTCTGCGAAAGGACTTGG-3′ (reverse).

### 2.6. Protein Extraction

The dorsal skin was collected from mouse, and the subcutaneous tissue was removed by scraping the isolated skin tissue. Tissue was then immediately frozen in liquid nitrogen. Samples were pulverized in liquid nitrogen using mortar and pestle. The pulverized tissue powder was immersed in SDS buffer (4% SDS, 20% Glycerol, 125 mM Tris pH 6.8) for 2 h in room temperature. Insoluble pellets were removed by centrifugation (17,700× *g* for 15 min).

### 2.7. Proteomic Analysis

Proteins in SDS buffer were processed with S-Trap column (Protifi, Farmingdale, NY, USA) according to the manufacturer’s instructions in order to obtain purified trypsin-digested peptides. Each sample was solubilized with phase transfer surfactant (PTS) buffer [17] and boiled at 95 °C for 5 min. The lysates were further sonicated with a Bioruptor sonicator (Cosmo Bio, Tokyo, Japan). Then, the samples were reduced with 10 mM TCEP; alkylated with 20 mM iodoacetamide; and sequentially, digested with trypsin (protein weight: 1/50) and Lys-C (protein weight: 1/50) for 16 h at 37 °C [18]. Peptides were desalted on a C18 StageTips. To quantify protein expressions, TMT labeling was conducted according to the manufacturer’s protocol. Labeled peptides by TMTpro 18plex reagents were fractionated into seven fractions using off-line basic pH reversed-phase (BPRP) fractionation. The proteomic analysis was conducted with an Orbitrap Fusion Lumos mass spectrometer (Thermo Fisher Scientific) coupled with Ultimate 3000 (Thermo Fisher Scientific) and an HTC-PAL (CTC Analytics). Peptides were delivered to an analytical column (75 μm × 30 cm, packed in-house with ReproSil-Pur C18-AQ, 1.9 μm resin) and separated at a flow rate of 280 nL/min using a 105 min gradient from 5% to 30% of solvent B (solvent A, 0.1% FA; solvent B, 0.1% FA and 99.9% acetonitrile). The settings used for the Orbitrap Fusion Lumos were similar to those described in a previous phosphoproteomic study [19]. Peptide identification was carried out with MaxQuant 1.6.14.0 supported by the Andromeda search engine. The UniProt database for mouse proteins (release 2020_03) combined with 262 common contaminants was used to analyze the LC-MS/MS data. The enzyme specificity was set to trypsin/P (the C-terminal of Arg or Lys with cleavage at the proline bond allowed). Instances of incorrect cleavage of up to two sites were tolerated. A fixed modification of carbamidomethylation of cysteine residue was assumed while methionine oxidation and serine, threonine, and tyrosine phosphorylation were set to variable modifications. The false discovery rate (FDR) of protein groups, peptides, and phosphosites was less than 0.01. Peptides that were identified from reversed database or identified as potential contaminants were not used in the following analysis. The statistical analysis was carried out with Perseus 1.6.14.0. The quantitative TMT reporter ion intensities were log2 transformed and normalized by median centering of the values in each TMT channel. Imputation of the missing value was not conducted. *T*-values were calculated using limma and *p*- and adjusted *p*-values were calculated using fdrtool.

### 2.8. Western Blotting

Proteins in SDS buffer were denatured at 95 °C for 5 min, separated by SDS-PAGE, transferred onto PDVF membranes, blocked with 2% milk in TBST for 30 min, and probed with anti-COL6A1 (GeneTex, Irvine, CA, USA, cat. GTX109963, 1:1000) or anti-Vinculin (Sigma, St. Louis, MO, USA, cat, V9131, 1:1000). After washing four times with TBST, membranes were incubated with 1:2000 diluted HRP-conjugated secondary antibodies (Cytiva, Emeryville, CA, USA) for 30 min at RT, washed four times again with TBST, and visualized with Amersham ECL reagents (Cytiva).

## 3. Results

### 3.1. Trancriptomic Effects of FA Treatment on the Skin

**Figure 1:**
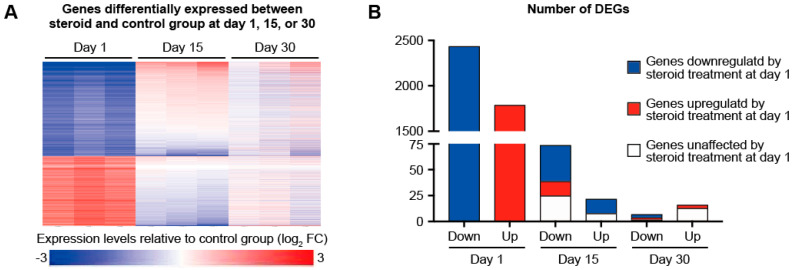
**Transcriptome of FA-treated skin returned close to normal by two weeks after the treatment.** (**A**) The heatmap represents the relative mRNA expressions of genes that were significantly differentially expressed between FA-treated and control skin samples at 1, 15, or 30 days after the treatments. (**B**) The bar graph shows the number of genes that were significantly differentially expressed between FA-treated and control skin samples at 1, 15, or 30 days after the treatments.

In order to investigate how chronic TCS treatment affects mouse skin at molecular levels, we shaved the dorsal hair of 7-week-old female C57BL/6 mice and applied FA (5 µg in 200 µL acetone) or vehicle once every 3 days for 12 days and then collected the skin samples 1, 15, or 30 days later. As expected, the skin samples collected 1 day after finishing 12 days of FA treatment exhibited reduced cell proliferation (Figure S1) and atrophy of epidermis and subcutaneous fat tissues (Figures S2 and S3). The skin samples collected 15 and 30 days after finishing 12 days of FA treatment were histologically recovered to the normal skin and no reduction was observed in cell proliferation (Figure S1) and the thickness of epidermis and subcutaneous fat tissues (Figures S2 and S3). To gain insights into the molecular events underlying the effects of chronic topical FA use, we first conducted bulk RNA-Seq analysis of the skin collected 1, 15, or 30 days after finishing 12 days of FA treatment (Table S1). The number of genes that were differentially expressed between control and FA-treated skin was by far the highest in the skin samples collected 1 day after finishing 12 days of FA treatment. At day 1, we identified 1791 up- and 2438 downregulated genes with at least two-fold expression changes with a *q*-value less than 0.05. Upregulated genes included *Fkbp5*, which is known to be induced by FA treatment [10], and we confirmed its upregulation by real-time qPCR (Figure S4A). At day 15, 22 upregulated and 74 downregulated genes were identified. At day 30, only 16 and 7 genes were upregulated and downregulated, respectively, in the FA-treated skin samples (Figure 1A,B). A majority of genes upregulated at day 15 after the 12 days of FA treatment were less expressed in FA-treated skin than in control skin at day 1 after the treatment. There were 14 such genes, of which 6 were those encoding keratins and keratin-associated proteins. This may suggest active proliferation of epithelial cells in FA-treated skin at day 15 after finishing 12 days of FA treatment.

**Figure 2:**
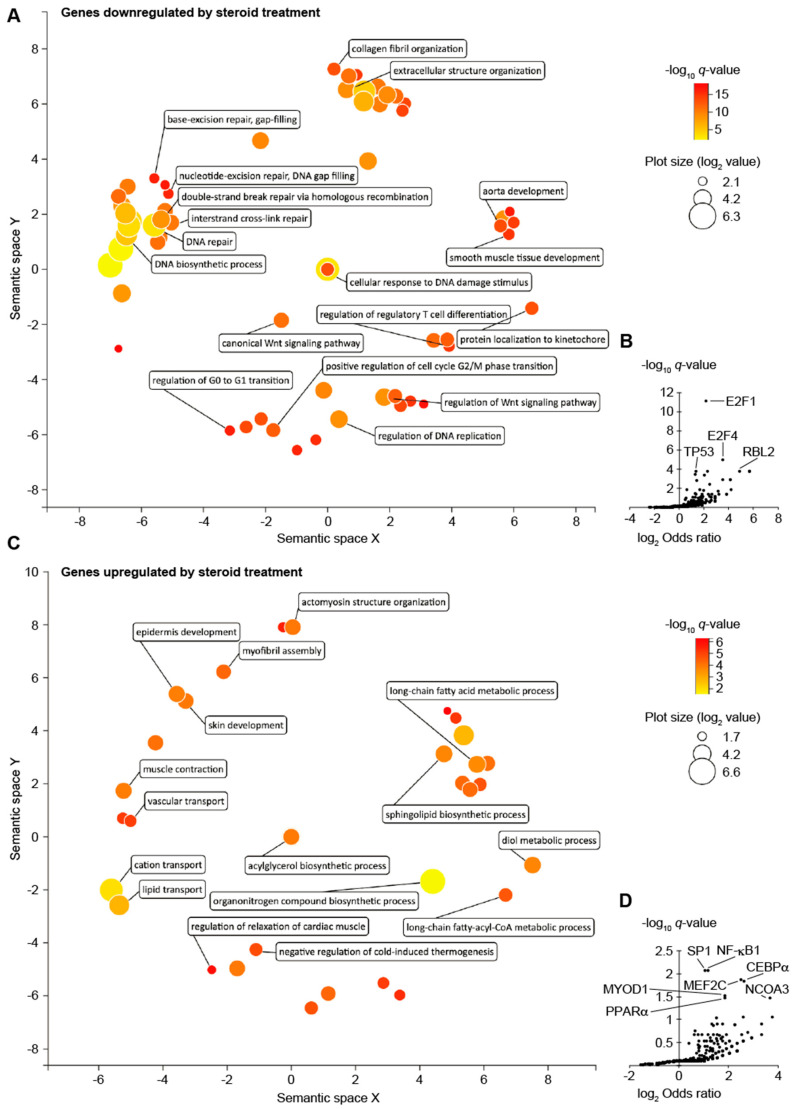
**The effects of FA treatment on the transcriptome of the mouse dorsal skin.** (**A**,**C**) GO biological process terms enriched in genes downregulated (**A**) and upregulated (**C**) in the skin 1 day after finishing 12 days of FA treatment. Ontology enrichment analysis was performed using Enrichr, and the results were visualized using REviGO. (**B**,**D**) Transcription factors (TF) whose target genes were enriched in genes downregulated (**B**) and upregulated (**D**) in the skin 1 day after finishing 12 days of FA treatment. TF target enrichment analysis was performed using TRRUST database and Enrichr.

GO ontology analysis showed that genes downregulated in the skin at 1 day after finishing 12 days of FA treatment were enriched with genes involved in DNA repair, cell-cycle progression, extracellular structure organization, aorta development, and Wnt signaling pathway (Figure 2A). Consistent with these observations, transcription factor (TF) target enrichment analysis using TRRUST indicated the target genes of cell-cycle regulator E2F1 were by far the most enriched TF targets (Figure 2B). Genes upregulated in the skin at 1 day after finishing 12 days of FA treatment were enriched with genes involved in lipid metabolic process, skin development, and myofibril assembly (Figure 2C). These genes were also enriched with the target genes of SP1, NF-κB, CEBPα, PPARα, and MYOD1 (Figure 2D).

### 3.2. Proteomic Effects of FA Treatment on the Skin

**Figure 3:**
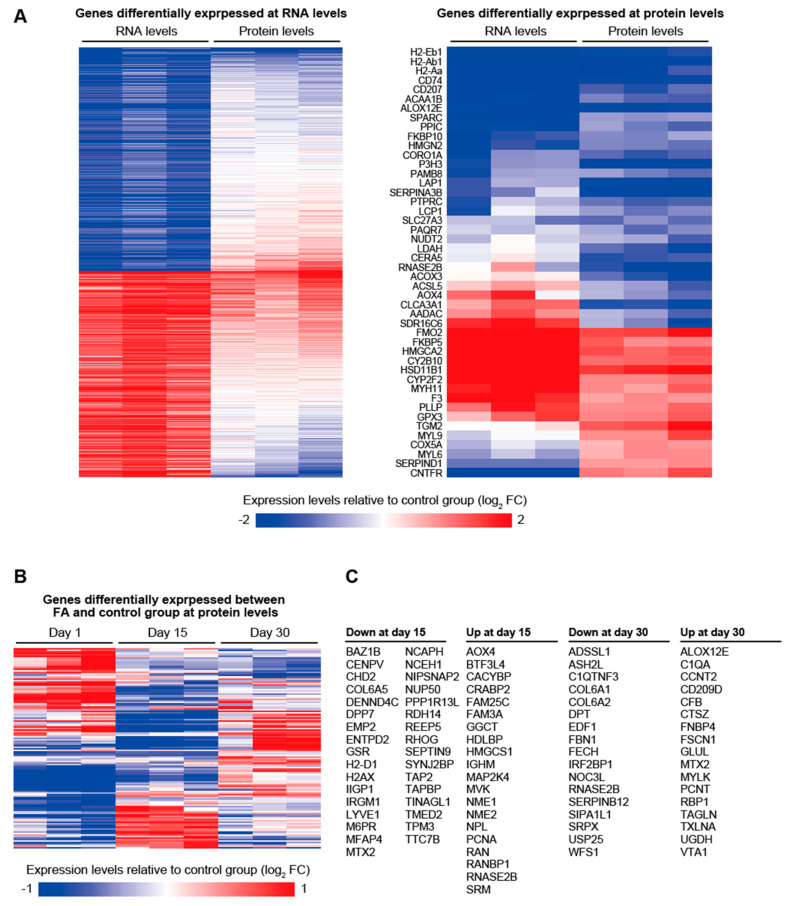
**The effects of FA treatment on the proteome of the mouse dorsal skin.** (**A**) The heatmaps represent the relative expression levels of genes in 1 day post-FA-treated skin samples compared to 1 day post-vehicle-treated skin samples. The left and right heatmaps show the relative expression levels of genes whose expression was significantly affected by FA treatment at RNA and protein levels, respectively. (**B**) The heatmap represents the relative protein expressions of genes that were significantly differentially expressed between FA-treated and control skin samples at 1, 15, or 30 days after the treatments. (**C**) List of genes that were significantly differentially expressed between FA-treated and control skin samples at 15 or 30 days after the treatments.

Compared to proteomic analysis, RNA-seq analysis is more sensitive and can provide more comprehensive information on gene expression profile. However, the expression change at RNA levels does not necessarily lead to expression change at protein levels, and the latter can occur without accompanying the former due to post-transcriptional regulations. Transcriptomic and proteomic data are therefore complementary, and their integration would lead to the better understanding of how gene expression changes are affected by TCS and how such gene expression changes mediate the effects of TCS treatment. Thus, we performed quantitative tandem-mass-tag (TMT) mass spectrometry analysis of the skin samples that were collected 1, 15, and 30 days after finishing 12 days-vehicle or FA treatment (Table S2). As a result, protein expression of 3387 genes was detected and quantified in all the samples. These 3387 genes included 547 genes for which mRNA expression was significantly affected in the skin at 1 day after finishing 12 days of FA treatment. Among these 547 genes, only 28 genes (5%) were found to be significantly differentially expressed at protein levels in the skin at 1 day after finishing 12 days of FA treatment (*q* < 0.05) (Figure 3A). Among these 28 genes, the direction of gene expression changes at mRNA and protein levels were the same in 23 genes but were opposite in the other 5 genes. A total of 46 genes were found to be significantly differentially expressed at protein levels in the skin at 1 day after finishing 12 days of FA treatment, indicating that only 50% (23/46) of these protein expression changes accompanied consistent mRNA expression changes (Figure 3A). This inconsistency might be partially due to the fact that subcutaneous tissue was removed for proteomic analysis but not for transcriptomic analysis. However, subcutaneous tissue has much lower cellularity (and RNA) compared to dermis and epidermis, and thus, we consider that the difference in the sample preparation would not significantly affect our conclusions. Histocompatibility genes (H2-Eb1, H2-Ab1, and H2-Aa), dendritic cell marker CD207, macrophage migration inhibitory (MIF) receptor CD74, procollagen folding machinery FKBP10, and collagen assembly-associated proteins SPARC and P3H3 were downregulated by FA treatment both at mRNA and protein levels, suggesting suppression of immune response and collagen fibril formation. Genes upregulated by FA treatment both at mRNA and protein levels included FKBP5 and HSD11B1. FKBP5 is already known to be upregulated by FA treatment [10] and importantly has been shown to mediate the atrophy-inducing effect of FA, possibly by suppressing Akt and mTOR [11]. HSD11B1 is an enzyme that activates GCs and has been reported as a critical driver of skin aging [20]. On the other hand, SDR16C6 that has been reported to be involved in the hair-follicle cycle and in the maintenance of sebaceous and meibomian glands [21] was upregulated at mRNA levels but was downregulated at protein levels. This suggests that FA treatment induces hair-follicle fragility at least partially by downregulating SDR16C6 at post-transcriptional levels.

Proteins whose expression was significantly affected by FA in the skin at 15 days after finishing FA treatment were different from those identified in the skin at 1 day, except for AOX4 and RNASE2B, which were downregulated at day 1 but then upregulated at day 15 (Figure 3B,C). Moreover, protein expression changes induced by FA at 1 and 15 days after finishing FA treatment showed significant negative correlation (Pearson correlation test, *p* = 0.027), which may reflect molecular processes that reverse the effect of FA treatment. A total of 53 proteins were found to be significantly differentially expressed in the skin collected 15 days after finishing FA or vehicle treatment. At day 15, COL6A5 and MFAP4 were downregulated, and PCNA was upregulated in FA-treated skin compared to control skin. MFAP4 plays roles in microfibrillar assembly and maturation of elastic fibers, and its reduction in photoaged skin has been shown to contribute to ECM degradation and reduced elasticity [22]. Upregulation of PCNA, on the other hand, suggests that epithelial cells are actively proliferating to recover the atrophied skin. At day 30, differences in protein expressions between control and FA-treated skin become smaller than at day 1 and 15, but still a substantial number of proteins (34 proteins) exhibited differential expressions (Figure 3B,C). Notably, protein expression changes induced by FA at 30 days after the treatment were again very different from those identified in 1 and 15 days post-treated skin. At day 30, COL6A1 and COL6A2 were downregulated in FA-treated skin compared to control skin, suggesting the role of collagen 6 in the long-term effect of chronic FA treatment. Western blotting confirmed that COL6A1 was downregulated in the skin at 30 days after finishing 12 days of FA treatment (Figure S4B). None of the FA-dependent proteins identified at day 15 and 30 were significantly differentially expressed at mRNA levels between control and FA-treated skin. Thus, although FA-treated skin reverted to normal histology by 15 days, expression of proteins, including those with known importance in skin physiology, kept changing even 2 weeks after stopping FA treatment, implying that expressions of many proteins are post-transcriptionally regulated during recovery from FA treatment.

## 4. Discussion

**Figure 4:**
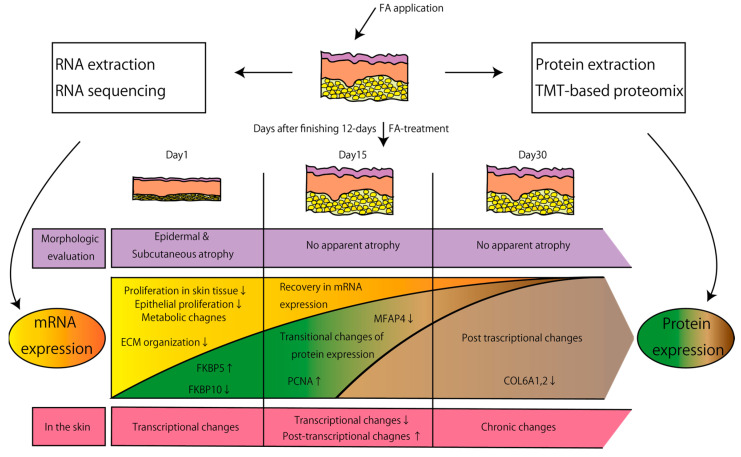
A summary of the current study.

Our transcriptomic and proteomic analyses provide a comprehensive overview of how gene regulations are affected after FA treatment in the mouse skin (Figure 4). FA is a glucocorticoid that is widely used in clinical practice, and its 2-week topical application has been shown to induce epidermal hypoplasia in the mouse skin to an extent similar to that observed in the human skin treated for 2 weeks with clobetasol propionate, one of the most frequently clinically used steroid [10]. While it remains largely unclear to what extent our findings apply to human skin, we found that many genes (*BEST2*, *CYP4B1*, *DDIT4*, *FKBP5*, *GALNT15*, *GLUL*, *PDK4*, *ZBTB16*) that were most strongly induced by one-time topical steroid application in human skin at 1 day after the treatment were also induced in mouse skin by 12 days of FA treatment at 1 day after the treatment [23].

As expected, our RNA-Seq data showed that genes involved in cell proliferation and genes regulated by E2F1 were preferentially downregulated in the skin 1 day after finishing 12 days of FA treatment (Figure 2A). Expression of these genes was recovered by 2 weeks after finishing FA treatment (Figure 1A), which is consistent with our histological observations. Several keratin and keratin-associated genes that were downregulated 1 day after finishing 12 days of FA treatment became more expressed in the FA-treated skin than in the control skin at 15 days after stopping the treatment, suggesting active epithelial development in the FA-treated skin at this point. Proliferation of epithelial cells may be also more active in 15-day post-treated skin than in the control skin, considering that PCNA was upregulated at protein levels at that point.

Thus, skin atrophy and cell proliferation returned almost to normal by 15 days after finishing 12 days of FA treatment, although some keratin, keratin-associated genes, and the cell proliferation marker PCNA were more expressed in the FA-treated skin at that point (Figure 4). In contrast to this, ECM and ECM-related gene expressions showed dynamic changes at protein levels at least up to 30 days after finishing 12 days of FA treatment. Interestingly, collagen 6 proteins were the only collagen proteins that were detected by mass spectrometry analysis to be downregulated in the FA-treated skin. At 1 day after finishing 12 days of FA treatment, none of the collagen proteins were differentially expressed, but Fkbp10 that has been reported to promote collagen 6 synthesis was downregulated in the FA-treated skin [24]. Collagen-associated proteins, namely SPARC and P3H3, were also downregulated at this point. COL6A5 and MFAP5, whose reduction in photoaged skin triggers ECM degradation [22], were downregulated 15 days after finishing 12 days of FA treatment. At day 30, COL6A5 expression was no longer affected but COL6A1 and COL6A2 expressions were downregulated in the FA-treated skin (Figure 4). Collagen 6 has been shown to be a key regulator of dermal matrix assembly, composition, and fibroblast behavior [25], and thus, our results suggest that long-term side effects of TCSs might be mediated by collagen 6 downregulation. Our results thus illustrate molecular changes underlying clinical efficacy and side effects of TCSs and would be a useful resource for future research.

## Data Availability

Unprocessed data are provided in Supplementary Materials.

## Supplementary Materials

The following supporting information can be downloaded at https://www.mdpi.com/article/10.3390/biom12121822/s1, Figure S1: Epidermal cell proliferation after finishing 12-days FA treatment; Figure S2: Atrophy of epidermis after finishing 12-days FA treatment; Figure S3: Atrophy of subcutaneous fat after finishing 12-days FA treatment; Figure S4: Validation of transcriptomic and proteomic analyses; Table S1: Normalized RNA-Seq data; Table S2: Normalized proteomic data.
