# Supplementary material for: Characterization of Transcriptomic and Proteomic Changes in the Skin after Chronic Fluocinolone Acetonide Treatment
Source: Biomolecules. 2022 Dec 6;12(12):1822. doi: 10.3390/biom12121822 (PMC9775701; doi:10.3390/biom12121822)
Supplement: Supplementary file 1 [file biomolecules-12-01822-s001.zip › Supplementary Figures.pdf]

Supplementary Information

Characterization of transcriptomic and proteomic changes in the skin after chronic fluocinolone acetonide treatment

Yongsu Choi, Masaki Takasugi, Kazuaki Takemura, Yuya Yoshida, Kamiya Tomonori, Jun Adachi, Daisuke Tsuruta, Naoko Ohtani

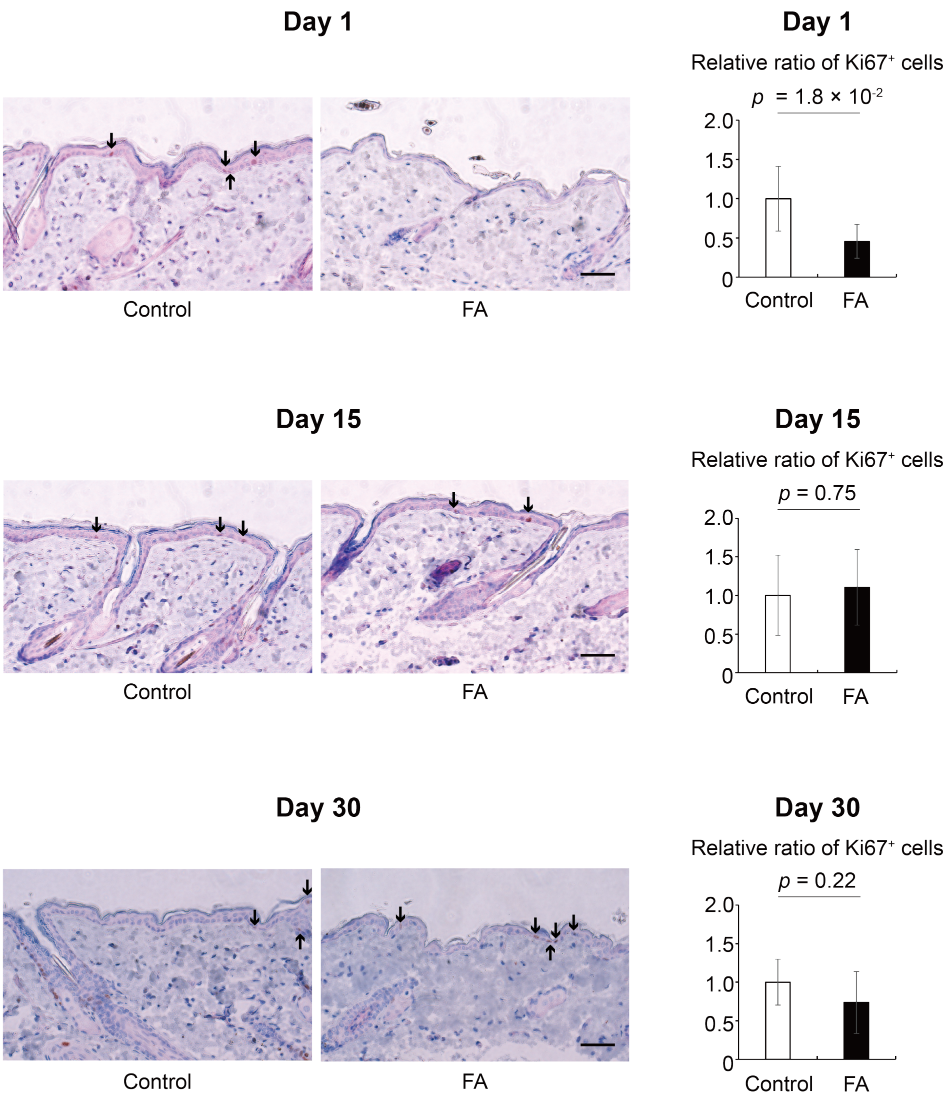

Figure S1. Epidermal cell proliferation after finishing 12-days FA treatment. Representative images of skin sections at 1, 15, and 30 days after finishing 12-days FA treatment. Sections were immunostained with Ki67 antibody and were counterstained with HE. Cells were counted at least for 4 fields of view for each sample and Ki67 positive ratio was calculated as the number of Ki67 positive cells / epithelial cells (excluding hair follicle cells). The bar graphs show the relative ratio of $Ki67^{+}$ cells in the epidermis (n = 7 for day 1 and day 30 samples and n = 6 for day 15 samples, two-sided Student t-test). Error bars represent standard deviation. Scale bar = 50 μm.

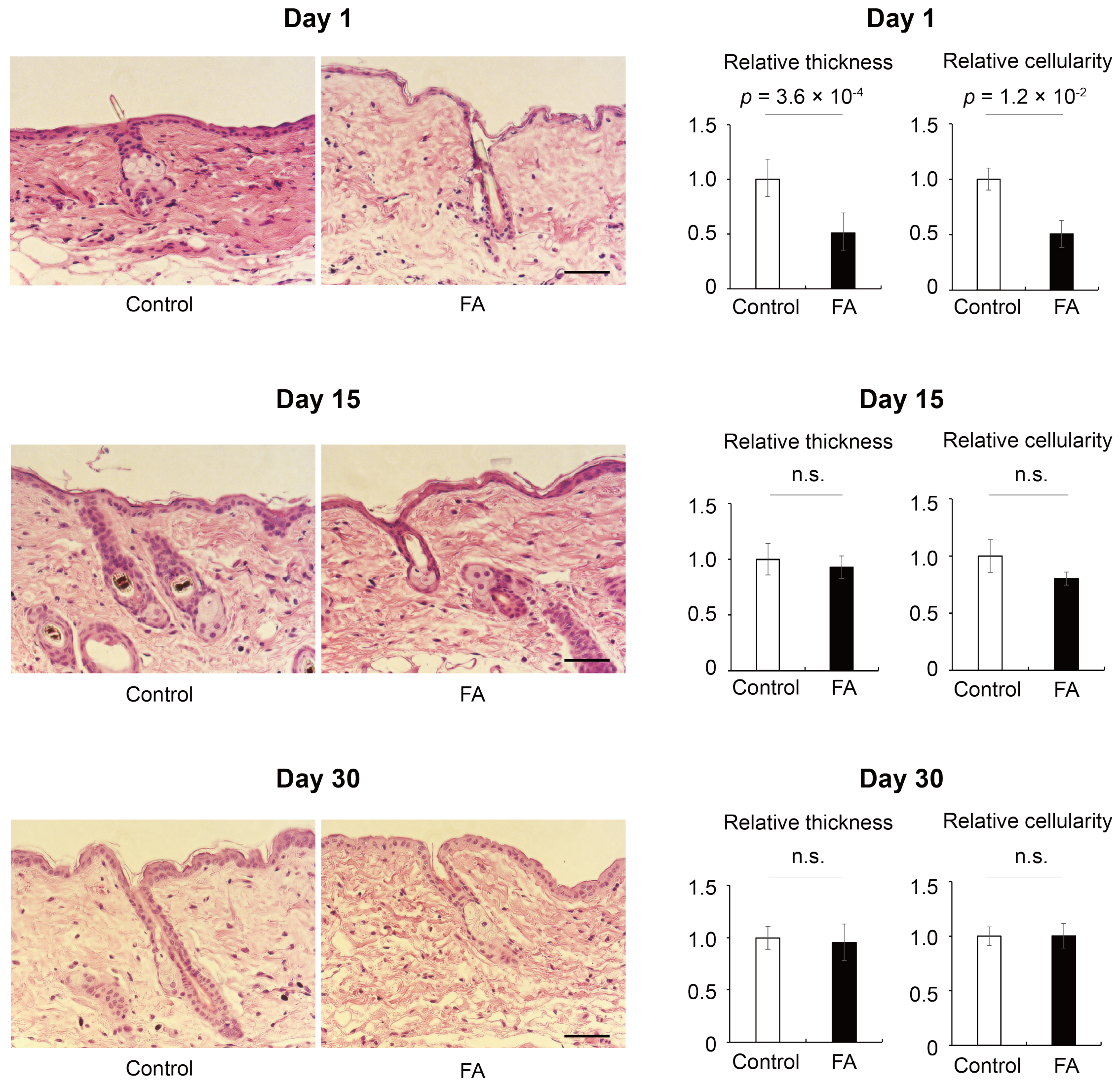

Figure S2. Atrophy of epidermis after finishing 12-days FA treatment. Representative images of skin sections at 1, 15, and 30 days after finishing 12-days FA treatment. Sections were stained with HE. The thickness of the skin was measured at more than 5 points and the number of cells was counted for 10 fields of views for each sample. The bar graphs show the relative thickness and cellularity of the epidermis (n = 3 for day 1 and 15 samples and n = 4 for day 30 samples, two-sided Student t-test). Error bars represent standard deviation. Scale bar = 50 μm.

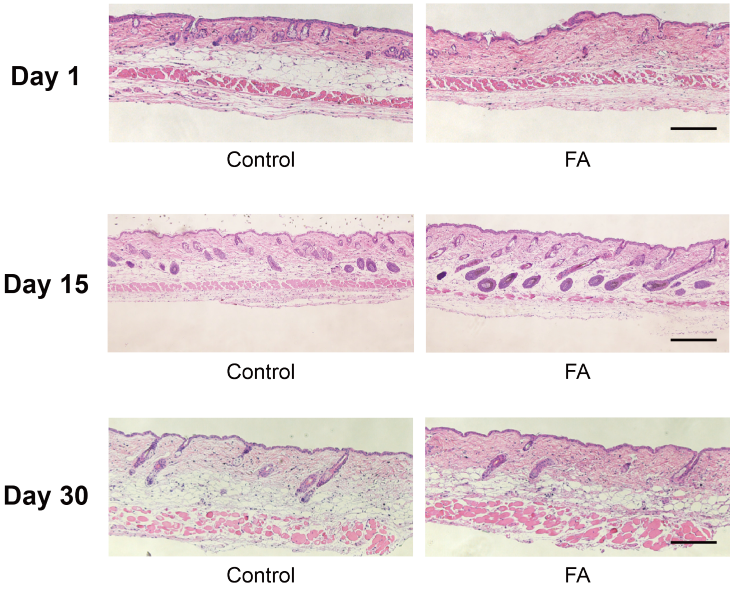

Figure S3. Atrophy of subcutaneous fat after finishing 12-days FA treatment. Representative images of skin sections at 1, 15, and 30 days after finishing 12-days FA treatment. Sections were stained with HE. Scale bar = 200 μm.

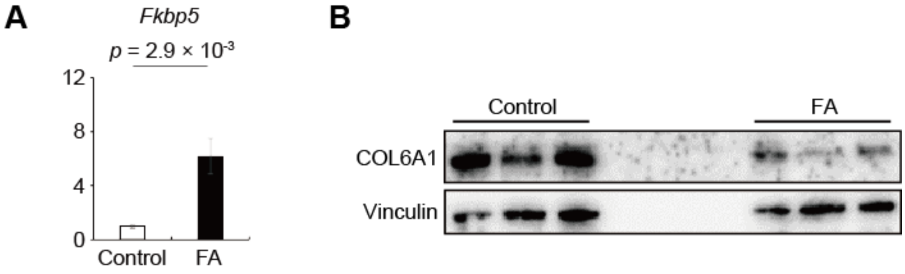

Figure S4. Validation of transcriptomic and proteomic analyses. (**A**) Expression levels of *Fkbp5* in the skin at 1 day after finishing 12-days FA-treatment. *Fkbp5* levels were measured by qPCR and were normalized to *Gapdh* levels (n = 3, two-sided Student t-test). Error bars represent standard deviation. (**B**) Immunoblotting of COL6A1 and Vinculin in the skin at 30 days after finishing 12-days FA-treatment (n = 3).
